# Hydroxysafflor Yellow A Protects Against Myocardial Ischemia/Reperfusion Injury *via* Suppressing NLRP3 Inflammasome and Activating Autophagy

**DOI:** 10.3389/fphar.2020.01170

**Published:** 2020-07-30

**Authors:** Jingxue Ye, Shan Lu, Min Wang, Wenxiu Ge, Haitao Liu, Yaodong Qi, Jianhua Fu, Qiong Zhang, Bengang Zhang, Guibo Sun, Xiaobo Sun

**Affiliations:** ^1^Beijing Key Laboratory of Innovative Drug Discovery of Traditional Chinese Medicine (Natural Medicine) and Translational Medicine, Institute of Medicinal Plant Development, Chinese Academy of Medical Sciences & Peking Union Medical College, Beijing, China; ^2^College of Pharmacy, Harbin University of Commerce, Harbin, China; ^3^Pneumology Department, Xiyuan Hospital, China Academy of Chinese Medical Sciences, Beijing, China

**Keywords:** myocardial ischemia reperfusion injury, hydroxysafflor yellow A, NLRP3, autophagy, AMPK

## Abstract

Myocardial ischemia/reperfusion (MI/R) injury is a serious threat to human health. Hydroxysafflor yellow A (HSYA), the main water-soluble ingredient extracted from Carthami flos (*Carthamus tinctorius* L.), has therapeutic potential for treating MI/R injury. However, the mechanisms of HSYA−mediated protection from MI/R injury are incompletely understood. In the present study, we investigated the effects and the underlying mechanisms of HSYA during MI/R. Adult Sprague-Dawley rats were subjected to left anterior descending artery ligation for 30 min followed by 24 h of reperfusion with or without HSYA treatment. The protective effect of HSYA was detected by 2,3,5-triphenyl tetrazolium chloride (TTC) staining, hematoxylin eosin (HE) staining, and myocardial enzymes detections. Serum levels of inflammatory factors such as TNF-α, interleukin (IL)-1β, and IL-18, were detected using ELISA kits. The expression of NLRP3 and other related proteins in the myocardium was detected by western blot and immunohistochemistry. The expression of autophagy-related proteins, including Atg5, BECN1, P62, and LC3B, was detected by western blot to evaluate the effect of HSYA on autophagy. Results showed that HSYA decreased the myocardial infarct size and attenuated the cardiac dysfunction in rats after I/R. In addition, HSYA inhibited myocardial apoptosis compared with the I/R group, decreased the levels of inflammatory cytokines in rat serum, reduced NLRP3 inflammasome expression, and induced autophagy. Mechanistically, our results demonstrated that HSYA can activate AMPK to improve autophagy and inhibit NLRP3 inflammasome by inhibiting the mTOR pathway. This work provides strong data supporting for the clinical applications of HSYA in MI/R injury.

## Introduction

Ischemic heart disease is the biggest threat to human health worldwide (Grant et al., 2017). Early blood flow recovery plays an important role in maintaining cardiac function and reducing myocardial cell damage in patients with myocardial ischemia. However, as blood flow is recovered, myocardial infarction caused by reperfusion injury can account for over half of the total infarct area caused by ischemia (Lesnefsky et al., 2017; Zheng et al., 2020). Therefore, the prevention and treatment of reperfusion injury is particularly critical.

The inflammatory response is one of the important pathological mechanisms of myocardial ischemia/reperfusion (MI/R) injury. Moreover, inflammatory response results in cardiomyocyte apoptosis during the acute phase. NLRP3 inflammasome has been fully characterized and plays an important role in MI/R injury. Reperfusion induces activation of NLRP3 inflammasome, and the resulting interleukin (IL)-1β, IL-18, and active caspase-1 are also involved in MI/R process (Toldo et al., 2019). Previous studies showed that inhibition or deletion of NLRP3 inflammasome could reduce myocardial infarction size and improve cardiac function in experimental animal models (Zuurbier et al., 2019). Autophagy, a lysosome-mediated pathway to degrade cell fragments and organelles, plays an important role in maintaining the balance of cell structure, metabolism, and function. Many studies have shown that autophagy of cardiomyocytes has a great influence in I/R injury and autophagy limits myocardial damage due to infarction (Bravo-San Pedro et al., 2017). Importantly, autophagy suppresses inflammasome assembly and negatively influences IL-1 and IL-18 secretion (De Biase et al., 2020). Thus, inhibiting NLRP3 inflammasome by regulating autophagy is an effective approach to alleviate MI/R injury.

Hydroxysafflor yellow A (HSYA), the main water-soluble ingredient extracted from Carthami flos (*Carthamus tinctorius* L.), is predominantly used in the treatment of cardiovascular diseases (Li et al., 2019; Xu et al., 2019). Previous study reported that HSYA can resist inflammation, alleviate oxidation and apoptosis, improve neurological function, alleviate infarct volume, inhibit thrombosis formation and platelet aggregation, reduce brain edema, and protect blood vessels (Han et al., 2016; Wang et al., 2016; Dong et al., 2017; Sun et al., 2018; Li et al., 2019). Recently, our laboratory found that HSYA has a protective effect on hypoxia/reoxygenation-induced cardiomyocyte damage *in vitro*, and the mechanism was related to NLRP3 inflammasome inhibition (Ye et al., 2020). However, whether HSYA could play a protective role in MI/R *in vivo* by inhibiting NLRP3 inflammasome has yet to be determined.

In the present study, we explore the cardiac protection of HSYA in an MI/R rat model *in vivo* and its potential mechanisms for NLRP3 inhibition. Our results provide strong data supporting for the clinical applications of HSYA in MI/R injury.

## Material and Methods

### Reagents

HSYA (purity > 98%) was produced by Shanghai Winherb Medical S&T Development (Shanghai, China). Diltiazem hydrochloride tablets (DTZ) ware obtained from Tianbian Pharmaceutical Co., Ltd. (Tianjin, China). Antibodies against P62 and LC3II were obtained from Sigma-Aldrich (St. Louis, MO, USA). Antibodies against NLRP3, ASC, caspase-1 were purchased from Abclonal (Wuhan, China). The primary antibodies against IL-1β, mammalian target of rapamycin (mTOR), and p-mTOR were obtained from Abcam (Cambridge, UK), and the primary antibodies against Bax, Bcl-2, and Caspase-3 were obtained from Proteintech (Wuhan, China). The primary antibodies against AMPK, p-AMPK, Atg5, and BECN1 were obtained from Cell Signaling Technology (Boston, USA).

### Animals

Adult male Sprague-Dawley rats (280±20 g) were obtained from Beijing Vital River Laboratory Animal Technology Co., Ltd. (Beijing, China). All animal experiments were conducted according to the principles of the Institutional Animal Care and Use Committee (IACUC) at the Chinese Academy of Medical Sciences and Peking Union Medical College (Beijing, China) (Approval No.: SLXD-20190702003). The animals were housed under controlled temperatures and humidity on a 12 h light-dark cycle. Food and water were provided *ad libitum*.

### Myocardial Ischemia/Reperfusion Model

Myocardial ischemia was inflicted by ligating the left anterior descending coronary artery (Xu et al., 2020). Briefly, 90 rats were anesthetized through intraperitoneal injection of pentobarbital sodium (40 mg/kg), intubated in the supine position, and monitored using electrocardiogram. After fur was removed, the thorax was opened, and the left anterior descending branch was ligated with a 6–0 suture for 30 min. The ligation was opened, and the heart was reperfused for 24 h. The sham group was subjected to the same procedures but without ligation. After the operation, the ventilator was removed when the rats were able to breathe spontaneously. They were then placed on a thermostatic blanket until they could walk, and were returned to the cage. After 24 h of reperfusion, the rats were weighed and anesthetized. Blood was obtained from the abdominal aorta, and the heart was removed and rinsed with phosphate-buffered saline (PBS). The hearts were randomly selected among the experimental group and placed in 4% paraformaldehyde for fixation, and the rest of the hearts were stored at –80°C. Whole blood obtained from the abdominal aorta was centrifuged (3,000 rpm, 15 min), and the supernatant serum was absorbed and frozen at –80°C for later use.

### Drugs Administration

Ninety rats were randomly divided into six experimental groups as follows: sham group (n=15), I/R group (n=15), 4 mg/kg HSYA group (n=15), 8 mg/kg HSYA group (n=15), 16 mg/kg HSYA group (n=15), and 16 mg/kg DTZ group (n=15) as a positive control. As a calcium antagonist, DTZ is widely used to treat coronary heart disease and hypertension. Clinical studies have shown that DTZ can increase coronary blood flow in patients with myocardial ischemia and prevent arrhythmias during reperfusion (Herzog et al., 1997; Pizzetti et al., 2001). In addition, several studies have shown that DTZ can reduce oxidative stress injury, correct energy metabolism, improve endothelial function, and regulate apoptosis (Peng et al., 2014; Chen et al., 2016). Therefore, DTZ was selected as the positive control in the present study. The dosage for HSYA and DTZ was chosen based on previous pharmacodynamics studies (Mu et al., 2017; Yu L. et al., 2018; Yu et al., 2020). HSYA and DTZ were dissolved in 0.9% sodium chloride and stored at 4°C. Rats in HSYA group were administered 4, 8, and 16 mg/kg HSYA by tail intravenous injection 30 min prior to ischemia operation. The same doses of HSYA were given 30 min after the onset of reperfusion. DTZ was orally administered to the rats once a day for five consecutive days prior to ischemia operation.

### Detection of Myocardium Infarct Size

The myocardial infarct size was assessed using 2,3,5-triphenyl tetrazolium chloride (TTC) (Sigma-Aldrich; Merck KgaA, Germany) as previously described (Wang R. et al., 2019). After all treatment, the heart was removed, rinsed with saline, and frozen using liquid nitrogen for 1 min. Along the heart axis, the heart was cut into six slices and incubated in 2% TTC for 15 min at 37°C in the dark, and then fixed with 4% paraformaldehyde for 24 h. The heart slices were photographed with a digital camera, and the proportion of the infarcted area was assessed from the obtained images.

### Detection of Myocardial Enzymes

The levels of creatine kinase-MB (CK-MB), aspartate transaminase (AST), and lactic dehydrogenase (LDH) in the serum were detected based on the commercial kits (BioSino, Beijing, China) using a Beckman AU480 biochemical auto-analyzer (Fullerton, CA, USA).

### Enzyme-Linked Immunosorbent Assay (ELISA)

The concentrations of IL-18, IL-1β, and TNF-α levels in the serum of rats were measured using commercial ELISA kits (Beijing Expandbiotech Ltd., Beijing, China) in accordance with the manufacturer's instructions.

### Histopathological Examination and Immunohistochemical Analyses

The heart tissue was fixed with 4% paraformaldehyde and embedded in paraffin. Subsequently, 5 μm sections were prepared and stained with hematoxylin and eosin (H&E). Then the pathological changes of heart tissue were detected using IncuCyte™ S3 ZOOM Cell Imaging System (Essen BioScience, Ann Arbor, MI, USA).

The immunohistochemical staining of NLRP3, ASC, and caspase-1 tissue sections was carried out according to the kit instructions. Briefly, the tissue sections were dewaxed and hydrated. Their antigen was retrieved, and the endogenous peroxidase was blocked. The sections were treated with goat serum and then incubated with NLRP3, ASC, and caspase-1 antibodies overnight. The slides were stained using the DAB kit, restained with hematoxylin, and observed using Aperio S2 Leica Biosystem microscope (Leica, Wetzlar, Germany). Quantification of all data was performed with ImageJ software.

### Determination of Myocardial Apoptosis

Terminal deoxynucleotidyl transferase-mediated dUTP nick end labeling (TUNEL) (Beyotime, Shanghai, China) was used to detect apoptosis in tissue sections in accordance with the reagent instructions. Briefly, the frozen sections were incubated with 1% Triton X-100 for 15 min at room temperature. The sections were incubated in proteinase K working fluid for 30 min, washed with PBS. TUNEL detection solution was added to the sample and incubated in the dark at 37° for 60 min. The sections were washed with PBS three times for 5 min each and incubated with 4′,6-diamidino-2-phenylindole (DAPI) to identify nuclei. Finally, antifading mounting medium was added to prevent fluorescence quenching. The images were acquired by Tissue Gnostics AX10 analysis system (Vienna, Austria).

### Western Blot Analysis

Heart tissues were lysed in radioimmunoprecipitation assay (RIPA) buffer (Beyotime, Shanghai, China) containing protease and phosphatase inhibitors. The protein concentrations were determined using the BCA assay (CWBiotech, Beijing, China); 40 μg of total proteins were loaded per lane, separated using 10% sodium dodecyl sulfate (SDS-PAGE), and blotted onto nitrocellulose membranes. The membrane was incubated overnight with the following primary antibodies: AMPK, p-AMPK, Atg5 and BECN1 (1:1,000, Cell Signaling Technology, USA), Bcl-2, Bax, caspase-3 and β-actin (1:1,000, Proteintech, China), IL-1β, mTOR, and p-mTOR (1:1,000, Abcam, UK), NLRP3, ASC and caspase-1 (1:1,000, Abclonal, China). The membranes were washed and incubated with the corresponding secondary antibodies. Finally, the bands were visualized using an ECL Kit (CW0049, CWBIO, Beijing, China).

### Statistical Analysis

Data are presented as the mean ± SEM. Statistical analyses were performed using GraphPad Prism 5.0., and one-way ANOVA followed by Tukey’s *post-hoc* test was used for multiple comparisons. The statistical significance was set at P < 0.05.

## Results

### HSYA Alleviates Myocardial Injury Caused by Myocardial Ischemia/Reperfusion in Sprague-Dawley Rats

**Figure 1:**
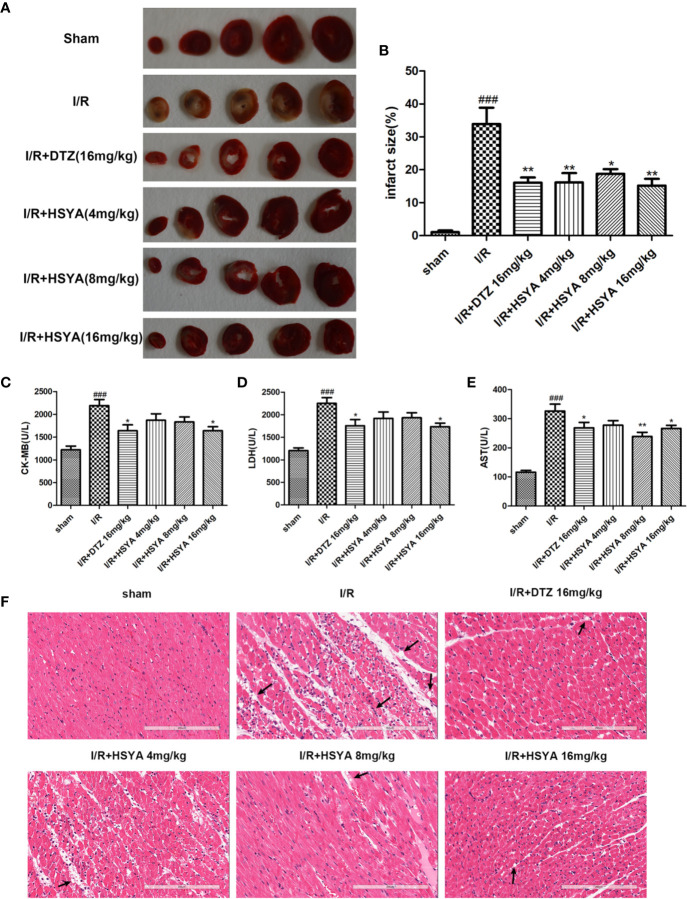
Hydroxysafflor yellow A (HSYA) alleviates I/R induced cardiac impairment. **(A, B)** The representative images and analysis results of triphenyl tetrazolium chloride (TTC) staining (n=4 per group). **(C–E)** Effects of HSYA on serum creatine kinase-MB (CK-MB), aspartate transaminase (AST), and lactic dehydrogenase (LDH) levels in the rats subjected to ischemia/reperfusion (I/R) injury (n=10 per group). **(F)** The representative images of H&E staining (Scale bar, 200 μm, n=4 per group). Data are presented as means ± SEM. *^###^P* < 0.001 *vs.* the sham group, *P < 0.05, **P < 0.01 *vs.* the I/R group.

To detect the effect of HSYA on myocardial infarction area, we performed TTC staining on cardiac tissues. As shown in **Figure 1A**, the infarcted areas were stained white and the uninfarcted areas were stained red. The observation showed that there was no infarction in the myocardial tissue of sham group, while significant infarction occurred in the myocardial tissue of I/R group. The areas of myocardial infarction in the HSYA groups were significantly reduced in a dose-dependent manner compared with the I/R group, and that 16 mg/kg HSYA was the most effective dose against MI/R injury, comparable to DTZ (**Figures 1A, B**).

The elevated levels of CK-MB, LDH, and AST in the blood can reflect the degree of myocardial tissue injury after MI/R. As shown in **Figures 1C–E**, the serum levels of CK-MB, LDH, and AST were obviously higher in the I/R group compared with sham group, which was decreased after HSYA and DTZ treatment.

Myocardial histomorphology was assessed by HE staining, and the results were shown in **Figure 1F**. The sham group exhibited normal structure without lesions, edema, or neutrophils. In contrast, obvious changes, such as myocardial structure disorder, neutrophils infiltration, interstitial edema, cardiomyocyte necrosis, and nuclear dissolution, were observed in the I/R group. After treatment with HSYA (4, 8, and 16 mg/kg) or DTZ, the structural disorder of cardiac muscle fibers improved and the arrangement of cell nuclei was orderly. Moreover, the numbers of infiltrated neutrophils and necrosis cells in HSYA and DTZ-treated groups were less compared with those in the I/R group. Those indicate that HSYA reduces the morphological changes of myocardial tissue and exerts protective effects after I/R injury.

### Hydroxysafflor Yellow A Alleviates Myocardial Apoptosis

**Figure 2:**
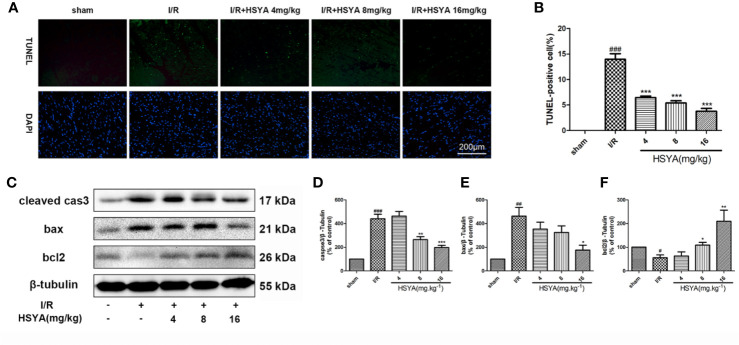
Hydroxysafflor yellow A (HSYA) reduces myocardial apoptosis. **(A, B)** Representative images and analysis results of terminal deoxynucleotidyl transferase-mediated dUTP nick end labeling (TUNEL) staining. Scale bar, 200 μm. **(C–F)** Representative immunoblots and quantified analysis of cleaved caspase-3, Bax, and Bcl-2 expression. Data are presented as means ± SEM (n = 3). *^#^P* < 0.05, *^##^P* < 0.01, *^###^P* < 0.001 *vs.* the sham group, *P < 0.05, **P < 0.01, ***P < 0.001 *vs.* the ischemia/reperfusion (I/R) group.

Accumulating evidence indicates that activation of cardiac apoptosis plays an important role in the pathophysiology of reperfusion injury (Teringova and Tousek, 2017). Apoptosis of cardiomyocytes was detected by TUNEL immunofluorescence staining. No significant myocardial apoptosis was observed in the sham group, but apoptosis cells significantly increased in I/R group compared with that in the sham group. HSYA dose-dependently reduced the number of TUNEL-positive cells, among the doses administered, 16 mg/kg HSYA was the most effective in reducing apoptosis (**Figures 2A, B**). The apoptosis-related proteins including cleaved caspase-3, Bax, and Bcl-2, were measured. The results showed that HSYA could inhibit the activation of caspase-3, reduce the expression of Bax and increase the expression of Bcl-2 (**Figures 2C–F**). Taken together, HSYA attenuates cardiac apoptosis in the myocardium of rats after I/R injury.

### Hydroxysafflor Yellow A Inhibits the Secretion of Inflammatory Factors

**Figure 3:**
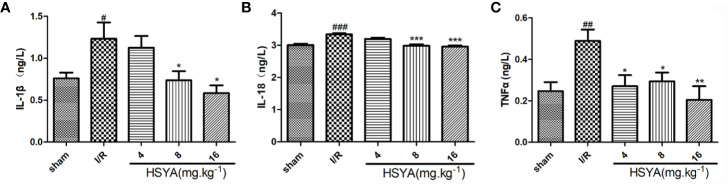
Hydroxysafflor yellow A (HSYA) inhibits the secretion of inflammatory factors. **(A)** Serum TNF-α level was analyzed by ELISA 24 h post-reperfusion (n=6 per group). **(B)** Serum interleukin (IL)-1β level was analyzed by ELISA 24 h post-reperfusion (n=6 per group). **(C)** Serum IL-18 level was analyzed by ELISA 24 h post-reperfusion (n=6 per group). Data are presented as means ± SEM. ^#^P < 0.05, ^##^P < 0.01, ^###^P < 0.001 *vs.* the sham group, *P <0.05, **P < 0.01, ***P < 0.001 *vs.* the ischemia/reperfusion (I/R) group.

Serum concentrations of inflammatory cytokines were determined by ELISA kits. As shown in **Figures 3A–C**, the serum levels of TNF-α, IL-1β, and IL-18 in the I/R group were significantly higher than those in the sham group. All HSYA groups ameliorated the excessive production of TNF-α, IL-1β, and IL-18 in serum induced by MI/R injury, indicating that HSYA reduces the inflammatory response after MI/R injury in rats.

### Hydroxysafflor Yellow A Inhibits the Expression of Inflammasome

**Figure 4:**
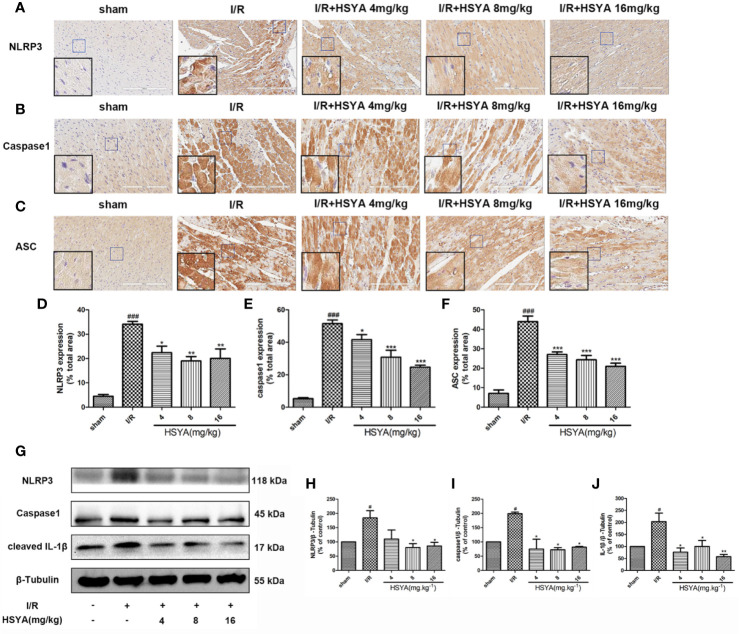
Hydroxysafflor yellow A (HSYA) inhibits the expression of NLRP3 inflammasome. **(A–C)** Immunohistochemical staining measuring NLRP3, ASC, and caspase-1 expressions in heart tissues of rats. **(D–F)** Quantification of NLRP3, ASC, and caspase-1 staining. **(G)** Western blot showing the protein expression levels of NLRP3, caspase-1, and cleaved interleukin (IL)-1β. **(H–J)** The ratios of NLRP3, caspase-1, and cleaved IL-1β and β-tubulin were quantified by densitometry based on immunoblot images. Data are presented as means ± SEM. *^#^P* < 0.05, *^###^P* < 0.01 *vs.* the sham group, *P < 0.05, **P < 0.01, ***P < 0.001 *vs.* the ischemia/reperfusion (I/R) group.

More studies have shown that the inflammatory response initiated by NLRP3 inflammasomes is involved in the pathophysiological process of MI/R (Toldo et al., 2018; Toldo et al., 2019; Zahid et al., 2019). To investigate the effects of HSYA on inflammasome, we examined the expression of NLRP3, ASC, and caspase-1 by immunohistochemistry. As shown in **Figures 4A–F**, NLRP3, ASC, and caspase-1 levels were increased in the I/R group compared with the sham group, whereas HSYA treatment decreased their levels induced by MI/R. Consistently, the expression of NLRP3, ASC, and caspase-1 proteins was detected by Western blot. Compared with the I/R group, HSYA significantly inhibited the expression of NLRP3, activation of caspase-1, and expression of IL-1β in a dose-dependent manner (**Figures 4G–J**).

### Hydroxysafflor Yellow A Activates Autophagy After Myocardial Ischemia/Reperfusion in Sprague-Dawley Rats

**Figure 5:**
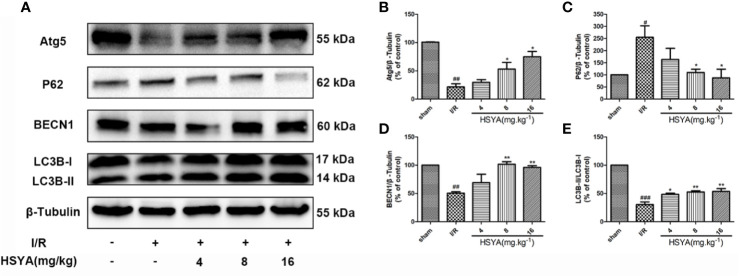
Hydroxysafflor yellow A (HSYA) induces autophagy in the myocardium. **(A)** Myocardial Atg5, P62, BECN1, and LC3B expressions were assayed by western blot analysis. **(B–E)** Quantification of protein expression. Data are presented as means ± SEM. *^#^P* < 0.05, *^##^P* < 0.01, *^###^P* < 0.001 *vs.* the sham group, *P < 0.05, **P < 0.01 *vs.* the ischemia/reperfusion (I/R) group.

Studies have shown that autophagy is an important process in ischemic heart disease, and autophagy flux is partially impaired and autophagosome clearance is significantly inhibited during reperfusion (Aghaei et al., 2019; Yang M. et al., 2019; Wang Y. et al., 2019). The conversion of LC3 I to LC3 II form is recognized as an indicator of autophagy activation. BECN1 is up-regulated and P62 is down-regulated in autophagy, which have important functions in autophagy regulation and are commonly used markers for autophagy detection. The expression of autophagy related proteins in the myocardial tissues was detected by western blot. As shown in **Figures 5A–E**, compared with the sham group, the LC3-II/LC3-I ratio and BECN1 and Atg5 expression in the I/R group significantly decreased, while the expression of P62 significantly increased in the I/R group, indicating that autophagy decreases during MI/R. HSYA reversed these changes and increased autophagy during reperfusion.

### Hydroxysafflor Yellow A Regulates AMPK/mTOR Signaling After Myocardial Ischemia/Reperfusion in Sprague–Dawley Rats

**Figure 6:**
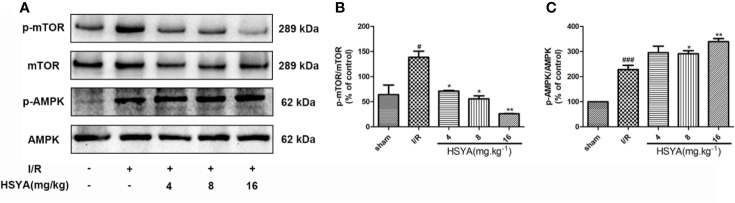
Hydroxysafflor yellow A (HSYA) attenuates myocardial ischemia/reperfusion injury by regulating the AMPK/mTOR pathway. **(A)** Protein expression levels of p-AMPK, AMPK, p-mTOR, and mTOR in heart tissues were assayed by western blot analysis. **(B, C)** Quantification of protein expression. Data are presented as means ± SEM. *^#^P* < 0.05, *^###^P* < 0.001 *vs.* the sham group, *P < 0.05, **P < 0.01 *vs.* the ischemia/reperfusion (I/R) group.

The AMPK/mTOR signaling is the key element for autophagy regulation and cardiac survival (Tamargo-Gómez and Mariño, 2018; Dong et al., 2019). During MI/R, the AMPK/mTOR signaling pathway modulates mitochondrial function, contractile function, autophagy, and apoptosis (Dake and Lawrence, 2015; Shi et al., 2018; Huang et al., 2018). To explore the effect of HSYA on the AMPK and mTOR proteins during MI/R in rats, we examined the levels of these proteins by Western blot. As shown in **Figures 6A–C**, compared with the sham group, the phosphorylation level of mTOR was significantly increased and AMPK was obviously reduced in the I/R group, whereas HSYA inhibited the phosphorylation of mTOR and increased the phosphorylation level of AMPK.

## Discussion

This study assesses the protective effect of HSYA on MI/R injury. Our study indicated that HSYA significantly reduces MI/R injury, inhibits myocardial inflammatory response, and reduces myocardial apoptosis. Consistent with our previous results on the cardiomyocyte injury model induced by hypoxia/reoxygenation, the myocardial protective mechanism of HSYA in the rat MI/R model may involve the promotion of myocardial autophagy by regulating AMPK/mTOR signaling pathway, inhibition of the activation of NLRP3 inflammasome, and reduction of the expression of downstream inflammatory factors to protect the ischemic myocardium.

Although early reperfusion plays an important role in improving myocardial ischemia, the recovery of blood flow could cause further damage to the ischemic tissue, namely reperfusion injury, resulting in the increase of myocardial infarction area. Many heart protection strategies have been tested on experimental animals over the past few decades, but clinical results have been disappointing. Moreover, no consensus on treatment is available (Hausenloy and Yellon, 2016). Therefore, it is necessary to further explore new therapeutic strategies to alleviate MI/R injury. HSYA has been widely used in the treatment of cerebrovascular and cardiovascular diseases. We discussed the protective effect of HSYA on MI/R injury in this study. The areas of myocardial infarction in the HSYA groups were significantly reduced in a dose-dependent manner compared with the I/R group. The elevated levels of CK-MB, LDH, and AST in the blood can reflect the degree of myocardial tissue injury after MI/R (Zhang et al., 2017). Our data showed that the serum levels of CK-MB, LDH, and AST are obviously higher in the I/R group than in the sham group, whereas HSYA inhibits these levels in a dose-dependent manner. Combined with the pathologic findings of the heart by HE staining, these results confirm the protective role of HSYA in the MI/R challenge.

Activation of myocardial apoptosis is a key feature of the pathology of MI/R injury, leading to myocardial cell loss and the subsequent inflammatory cell infiltration and inflammatory response (Xia et al., 2016; Teringova and Tousek, 2017; Toldo et al., 2018; Soares et al., 2019). It is well known that Bcl-2 and Bax are important proteins in cell apoptosis. In addition, caspase-3 is a key protein in the apoptotic pathway and plays a role when processed into cleaved caspase-3 (Zhou et al., 2019). In the present study, our results confirmed that HSYA can down-regulate the expression of pro-apoptosis proteins including Bax and cleaved caspase-3 and up-regulate the expression of anti-apoptosis protein Bcl-2 in MI/R injury. Meanwhile, TUNEL staining indicated that HSYA decreases I/R-induced apoptosis of cardiomyocytes in rats.

Inflammation is a major cause of MI/R injury (Ong et al., 2018). Recent studies have indicated that NLRP3 inflammasome plays a critical role in the development of heart inflammation (Shao et al., 2015; Liu et al., 2018). When activated, NLRP3 and ASC form inflammasome complexes that control the activation of caspase-1 (Sun et al., 2017; Zahid et al., 2019). Subsequently, NLRP3 processes the secretion of inflammatory cytokines, including IL‐1β and IL‐18 (Deretic and Levine, 2018). Therefore, inhibition of NLRP3 activation can inhibit the secretion of inflammatory factors, thereby reducing myocardial injury caused by I/R (Xu et al., 2020; Wu et al., 2020). In view of the important role of NLRP3 in MI/R injury, we examined the effect of HSYA on NLRP3 inflammasomes and the secretion of inflammatory factors. Our experimental results suggested that HSYA decreases the release of inflammatory cytokines IL‐1β and IL‐18, and effectively downregulates the expression of NLRP3, ASC, and caspase-1 in I/R-induced rats.

Autophagy regulation is believed to be related to cardiovascular disease, and plays an important role in MI/R. It has been shown that moderate autophagy upregulation can reduce the degree of myocardial ischemia injury and cardiac dysfunction (Aghaei et al., 2019). Moreover, studies have shown that autophagy is closely related to immune cellular response and inflammatory pathways (Castillo et al., 2012; Sarah et al., 2013; Yu M. et al., 2018). Increasing evidence indicates that autophagy activation inversely regulates the activation of NLRP3 inflammasome and the secretion of inflammatory cytokines (Nakahira et al., 2011; Harris et al., 2017; Liu et al., 2018). Although HSYA has been shown to inhibit NLRP3 inflammasome, it remains to be demonstrated whether autophagy is involved in HSYA heart protection *in vivo*. The current study’s results revealed that HSYA promoted autophagy in cardiomyocytes, showing that it up-regulated autophagy protein expression of BECN1, Atg5, and LC3II and down-regulated expression of P62.

The pathogenesis of MI/R injury is complicated and involves multiple mechanism. Apoptosis, autophagy, and inflammation interact to influence the pathological process of MI/R (Dominic et al., 2019; Dong et al., 2019). Excessive apoptosis and inflammation aggravate MI/R injury (Dong et al., 2019). In contrast, moderate autophagy protects the myocardium against I/R injury (Aghaei et al., 2019). Inhibition of autophagy has been shown to promote inflammation *via* inflammasome activation (Liu et al., 2018). In addition, apoptosis and autophagy have a complex interplay and interact with each other in MI/R. Some pathways such as mTOR and AMPK induce the interplay between apoptosis and autophagy (Huang et al., 2018; Dong et al., 2019). Furthermore, inflammatory response aggravates myocardial apoptosis. Studies have shown that cardiomyocyte-specific transgenic overexpression of procaspase-1 was sufficient to activate caspase-3 and exacerbate apoptosis and infarct size in response to I/R (Faisal et al., 2005). Therefore, drug targeting multiple mechanisms may be a promising therapy for MI/R. In this study, we demonstrated HSYA protected against MI/R through multiple pathways including promoting myocardial autophagy and inhibiting the inflammation and apoptosis.

To further investigate the underlying mechanisms of HSYA cardiac protection, we examined the role of HSYA in the AMPK-mTOR pathway which is an important pathway for regulating autophagy and NLRP3 inflammasome. The mTOR pathway is a well-known negative regulator of autophagic activity that has been established to regulate cell growth, proliferation, and metabolism (Galluzzi et al., 2017; Rabanal-Ruiz et al., 2017; Shi et al., 2018). Furthermore, mTOR has an accelerating effect on the metabolic inflammation response (Moon et al., 2015). Consistent with previous researchers, our data indicated that the expression of mTOR is enhanced in the MI/R rats, but downregulated by HSYA. AMPK senses cellular energy status to maintain energy homeostasis. Evidence supports the role for AMPK in autophagy induction in response to various cellular stresses (Alers et al., 2011; Kim et al., 2011; Tamargo-Gómez and Mariño, 2018; Wang Y. et al., 2019). Notably, AMPK activation attenuates NLRP3 inflammasome upregulation in some pathological processes, such as diabetes, pain, ischemic stroke, and endoplasmic reticulum stress (Yang F. et al., 2019; Qing et al., 2019). In our present study, HSYA increased the phosphorylation level of AMPK. Moreover, AMPK is known as the upstream of mTOR which play an important role in the regulation of inflammation and autophagy. However, autophagy is a complex dynamic process, and multiple signaling pathways are considered to trigger autophagy in MI/R, including Beclin-1/class III phosphatidylinositol-3 kinase (PI3K), AMPK/mTOR, and PI-3K/Akt/mTOR pathways (Wang et al., 2015; Lin et al., 2018). Whether HSYA regulates autophagy through other signaling pathways requires further experimental investigation.

In summary, our present study reveals the underlying mechanism of HSYA against MI/R injury by promoting myocardial autophagy and inhibiting the activation of NLRP3 inflammasome through regulation of the AMPK/mTOR signaling pathway.

## Data Availability Statement

The raw data supporting the conclusions of this article will be made available by the authors, without undue reservation, to any qualified researcher.

## Ethics Statement

The animal study was reviewed and approved by the Institutional Animal Care and Use Committee (IACUC) at the Chinese Academy of Medical Sciences and Peking Union Medical College.

## Author Contributions

JY, GS, and XS conceived and designed the experiments. JY, SL, MW, WG, HL, and YQ performed the experiments. JF and QZ collected and analyzed the data. SL wrote original draft. BZ, GS, and XS revised the manuscript. BZ, GS, and XS supervised manuscripts. XS validated the manuscript, wrote review, and edited.

## Funding

This work was supported by National Natural Sciences Foundation of China (No. 81673817), and the Natural Sciences Foundation of Beijing (No. 7172191).

## Conflict of Interest

The authors declare that the research was conducted in the absence of any commercial or financial relationships that could be construed as a potential conflict of interest.
