# Corrigendum: Hydroxysafflor Yellow A Protects Against Myocardial Ischemia/Reperfusion Injury *via* Suppressing NLRP3 Inflammasome and Activating Autophagy

**DOI:** 10.3389/fphar.2021.671318

**Published:** 2021-04-22

**Authors:** Jingxue Ye, Shan Lu, Min Wang, Wenxiu Ge, Haitao Liu, Yaodong Qi, Jianhua Fu, Qiong Zhang, Bengang Zhang, Guibo Sun, Xiaobo Sun

**Affiliations:** ^1^ Beijing Key Laboratory of Innovative Drug Discovery of Traditional Chinese Medicine (Natural Medicine) and Translational Medicine, Institute of Medicinal Plant Development, Chinese Academy of Medical Sciences & Peking Union Medical College, Beijing, China; ^2^ College of Pharmacy, Harbin University of Commerce, Harbin, China; ^3^ Pneumology Department, Xiyuan Hospital, China Academy of Chinese Medical Sciences, Beijing, China

**Keywords:** myocardial ischemia reperfusion injury, hydroxysafflor yellow A, NLRP3, autophagy, AMPK

There is an error in the **Funding Statement**. The correct number for “National Natural Sciences Foundation of China” is “81673817”.

The authors apologize for this error and state that this does not change the scientific conclusion of the article in any way. The original article has been updated.

